# The effect of nasal *Staphylococcus aureus* colonization and antibiotic treatment on disease activity in ANCA-associated vasculitis: a retrospective cohort study in the Netherlands

**DOI:** 10.1007/s00296-022-05228-8

**Published:** 2022-10-26

**Authors:** Caroline M. Schaap, Roline M. Krol, Hilde H. F. Remmelts, Ruth Klaasen, E. Christiaan Hagen, Julia Spierings, Marloes W. Heijstek

**Affiliations:** 1 Department of Rheumatology and Clinical Immunology, University Medical Centre Utrecht, Utrecht, The Netherlands; 2 Department of Internal Medicine, Meander Medical Centre Amersfoort, Amersfoort, The Netherlands; 3 Department of Rheumatology, Meander Medical Centre Amersfoort, Amersfoort, The Netherlands

**Keywords:** S. aureus colonization, ANCA vasculitis, Cotrimoxazole, ENT involvement, Disease acivity

## Abstract

The aim of this study was to identify the role of nasal *Staphylococcus aureus* (*S. aureus*) colonization and the effect of systemic or local antibiotic treatment on disease activity in patients with antineutrophil cytoplasmic antibodies (ANCA) associated vasculitis and ear nose and throat (ENT) involvement. Clinical, laboratory and histological data from all patients with ANCA-associated vasculitis and ENT involvement, who were diagnosed in two medical centres in The Netherlands between 1981 and 2020, were retrospectively collected. Nasal *S. aureus* colonization was defined as at least one positive nasal swab during follow-up. Data on systemic (cotrimoxazole and azithromycin) and local (mupirocin) antibiotic use were collected. Disease activity was divided into systemic and local disease activity. Univariate analyses and regression analyses (negative binomial Poisson and binary regression) were used. Two-hundred and thirteen patients were available for analysis. Median follow-up time was 8 (IQR 3–17) years. *S. aureus* colonization was tested in 100 (46.9%) cases of whom 44 patients (44%) tested positive. In these 100 patients, systemic and local disease activity at baseline and at last visit were comparable between patients with and without *S. aureus* colonization. Twenty-eight of the 44 *S. aureus* positive patients received antibiotics aimed at eradication of *S. aureus*. No statistically significant difference was found between the treated versus non-treated group with regard to systemic and local disease activity. Nasal *S. aureus* colonization does not influence systemic or local disease activity. Antibiotic treatment aimed at eradication did not modify disease activity.

**Supplementary Information:**

The online version contains supplementary material available at 10.1007/s00296-022-05228-8.

## Introduction

Antineutrophil cytoplasmic antibodies (ANCA)-associated vasculitis (AAV) is a necrotizing vasculitis, predominantly affecting small or medium vessels with few or no immune deposits [1]. Presence of autoantibodies directed against neutrophil cytoplasmic constituents, predominantly proteinase 3 (PR3) and myeloperoxidase (MPO), is a hallmark of AAV [2].

AAV is subdivided into three subtypes; microscopic polyangiitis (MPA), granulomatosis with polyangiitis (GPA) and eosinophilic granulomatosis with polyangiitis (EGPA) [3].

The clinical characteristics can vary among these three subtypes. Both GPA and EGPA are characterized by necrotizing granulomatous inflammation often involving the respiratory tract. Ear nose and throat (ENT) involvement is most common in GPA [4, 5].

AAV has a relapsing—remitting disease course. Several risk factors for relapses have been reported, including bacterial infections especially nasal *Staphylococcus aureus* (*S. aureus*) infections in GPA [6–12] Some studies have shown a higher rate of chronic nasal colonization with *S. aureus* in GPA patients compared to healthy individuals [7, 10, 13, 14]. In contrast to the general population of which one-third has intermittent and one-third has chronic colonization of *S.aureus*, in GPA patients 60–70% is carrier [15]. Nasal *S.aureus* carriage is a global phenomenon. In The Netherlands an estimated 35% of the healthy population is colonized with *S.aureus* [16]. In some studies, nasal *S.aureus* colonization in AAV was associated with relapse of disease activity. This finding led to the use of antibiotics in AAV [17, 18].

However, the effect of antibiotics on disease activity in AAV patients with *S. aureus* colonization is controversial. Some studies showed earlier time to remission [17, 19] or prevention of relapses [9, 17, 20] in GPA patients when treated with cotrimoxazole. Other studies found no beneficial effect of cotrimoxazole on disease activity in GPA patients colonized with *S. aureus* [10, 13, 21]. Efficacy of other antibiotics than cotrimoxazole on disease activity, and the effect of antibiotics in *S. aureus* colonized EGPA and MPA patients are not known [17]. Therefore, the aim of this study is to determine the effect of nasal *S. aureus* colonization and treatment with local or systemic antibiotics on disease activity in AAV patients with ENT involvement.

## Methods

### Case definition

In this retrospective cohort study, we analysed the presence of nasal *S.aureus* colonization in patients with ANCA-associated vasculitis and ENT involvement. In case of *S.aureus* colonization, we analysed the effect of antibiotic treatment on disease activity.

Disease activity was divided into systemic and local disease activity. Systemic disease outcomes included history of one or more relapses, relapse number per patient year and BVAS3 score at last visit. Local disease outcomes included the development of saddle nose deformity or subglottic stenosis during follow-up and history of one or more ENT relapses.

### Data collection and participants

Data from patients with AAV from the University Medical Centre Utrecht  and Meander Medisch Centrum Amersfoort diagnosed between 1981 and 2020 were collected. Both centres are vasculitis referral centres. Patients were identified using related International Classification of Diseases (ICD) codes. AAV was defined by the Chapel Hill consensus criteria [3]. ENT involvement was defined as presence of at least one of the ENT symptoms stated in Birmingham vasculitis activity score (BVAS) version 3 (BVAS3) [22]. Saddle nose deformity or subglottic stenosis were defined as irreversible damage*.*

Clinical, laboratory and histopathology data were prospectively collected (during routine care patients visits) and retrospectively extracted from the electronic patient records. Two medical experts were consulted in case medical data appeared indistinct.

Clinical data included gender, disease duration, age at disease onset and last visit, ethnicity, comorbidities according to the Charlson comorbidity index [23], AAV type, ANCA status, organ involvement, disease activity defined by the BVAS3 at diagnosis and at last visit, number and characteristics of relapses [22]. Relapse was defined as a rise in BVAS3 score of at least one point, new or progression of existing symptoms or the need for treatment intensification [22]. No difference was made between major and minor relapse. The follow-up period was defined as the period between diagnosis and last clinic visit or death. Treatment related data that were collected included details concerning induction and maintenance therapy, dose, duration and administration route, maximum dosage of steroids and cumulative dose of cyclophosphamide.

Laboratory results were collected at diagnosis and at last visit within a time frame of 3 months at diagnosis and within a time frame of 6 months at last visit. These included ANCA-titre, C-reactive protein (CRP), erythrocyte sedimentation rate (ESR), leukocyte count, estimated glomerular filtration rate (eGFR), serum creatinine and the presence of protein in urine (proteinuria).

Histopathology data included results from tissue biopsies performed on ENT, kidney, lung, skin and/or other tissue. Results were divided into supportive, inconclusive or non-supportive for the diagnosis of AAV as concluded by the pathologist.

Data on ENT involvement included ENT symptoms according to BVAS3 score (either reported in patient records or calculated based on reported symptoms) at diagnosis and during follow-up and presence of irreversible damage (saddle nose deformity and subglottic stenosis). ENT limited AAV was defined as the presence of vasculitis activity in the nose without further systemic disease activity. Additionally, information with regard to *S. aureus* colonization and treatment with systemic antibiotics (cotrimoxazole and azithromycin), local antibiotics (mupirocin), and nasal steroids or nasal lavage with saline solution (NaCl 0.9%) was collected. Colonization was defined as at least one positive nasal swab during follow-up.

### Inclusion and exclusion criteria

Inclusion criteria were patients aged 18 years and older, with AAV defined by the Chapel Hill consensus criteria and ENT involvement according to BVAS3 score and a performed *S.aureus* colonization test. Exclusion criteria were patients without available data on ENT involvement or colonization.

### Statistical analysis

Descriptive tests were used for baseline characteristics of the study population. Categorical data were presented in numbers and percentages, continuous variables were described as median with interquartile ranges (IQR).

First, univariate analyses were used to analyse the influence of *S. aureus* on disease activity.

Presence of relapse was analysed by Pearson chi-square test and relapse number per patient year and BVAS3 score at last visit were analysed using Mann–Whitney *U* test.

Local disease activity, consisting of presence of ENT relapse, development of saddle nose deformity or subglottic stenosis during follow-up were analysed by Pearson Chi-Square tests.

Second, to correct for confounders gender, age at onset, AAV type, follow-up time and use of nasal steroids, regression analyses were performed to analyse the effect of *S. aureus* colonization on disease activity. The number of patients with subglottic stenosis at last visit was too small for regression analysis.

The number of relapses per patient year were analysed using a negative binomial Poisson regression because of non-normal distribution of data to calculate incidence rate-ratios of relapses during follow-up. Binary regression analyses were used to analyse the effect of *S. aureus* colonization on the presence of one or more ENT relapses during follow-up and the development of saddle nose deformity during follow-up presented as odds ratio (OR) with 95% confidence interval (CI).

To analyse the effect of *S. aureus* eradication, the same univariate analyses and systemic and local outcome measurements were used as mentioned above. Patient numbers were too small to perform regression analysis on the effect of antibiotic treatment on systemic and local disease activity.

*P* values ≤ 0.05 were considered statistically relevant. IBM SPSS Statistics version 25.0.0.2 was used.

## Results

### Baseline patient characteristics

A total of 213 AAV patients with ENT involvement were included. Baseline characteristics and treatment details of all included patients and patients with a performed test for *S. aureus* colonization are mentioned in Table 1. Median follow-up time was 8 (IQR 3–17) years. Use of nasal steroids during follow-up were prescribed to 37.6% of the patients.

**Table 1:** Baseline characteristics

Characteristics	*N* = 213 (total)	*N* = 100 (tested for *S. aureus* colonization)
Female,* n* (%)	116 (54.5%)	60 (60%)
ENT limited AAV, * n* (%)	36 (16.9%)	20 (20%)
Ethnicity, * n* (%)		
Caucasian	145 (68.1%)	75 (75%)
Asian	1 (0.5%)	1 (1%)
Other	6 (2.8%)	3 (3%)
Unknown	61 (28.6%)	21 (21%)
Age in years at onset of disease, median (IQR)	53 (40–62)	50 (35–60)
Age in years at last visit, median (IQR)	63 (51–74)	61 (48–73)
Deceased, * n* (%)	32 (15.0%)	14 (14%)
AAV type, * n* (%)		
GPA	178 (83.6%)	83 (83%)
EGPA	24 (11.3%)	12 (12%)
MPA	4 (1.9%)	1 (1%)
Unspecified	7 (3.3%)	4 (4%)
ANCA at diagnosis, * n* (%)		
PR3	127 (59.6%)	65 (65%)
MPO	32 (15.0%)	14 (14%)
Negative	34 (16.0%)	14 (14%)
Unknown	20 (9.4%)	7 (7%)
Biopsy performed, * n* (%)	173 (81.2%)	86 (86%)
Biopsy supporting AAV diagnosis, * n* (%)	101 (47.4%)	49 (49%)
BVAS3 at diagnosis, median (IQR)	13 (7–21)	12 (6–21)
BVAS3 at last visit, median (IQR)	0 (0–4)	0 (0–4)
Induction therapy		
Cyclophosphamide	143 (67.1%)	61 (61%)
Methylprednisolone pulse therapy	58 (27.2%)	29 (29%)
Methotrexate	20 (9.4%)	13 (13%)
Rituximab	46 (21.6%)	34 (34%)
Plasmapheresis	17 (8.0%)	4 (4%)
Mofetil mycophenolate	7 (3.2%)	0 (0%)
Azathioprine	4 (1.9%)	0 (0%)
IVIG	1 (0.5%)	0 (0%)
Cyclosporine	1 (0.5%)	0 (0%)
CCX168 (avacopan)	1 (0.5%)	1 (1%)
Omalizumab	1 (0.5%)	0 (0%)
Mepolizumab	1 (0.5%)	1 (1%)
Maintenance therapy		
Azathioprine	153 (71.8%)	75 (75%)
Mofetil mycophenolate	33 (15.5%)	20 (20%)
Rituximab	33 (15.5%)	17 (17%)
Methotrexate	22 (10.3%)	16 (16%)
Cyclophosphamide	1 (0.5%)	0 (0%)
Cyclosporine	2 (0.9%)	0 (0%)
Mepolizumab	4 (1.9%)	3 (3%)
Follow-up time in years, median (IQR)	8 (3–17)	8 (3–18)
Number of relapses per patient year, median (IQR)	0.1 (0–0.2)	0.1 (0–0.2)
History of one or more ENT relapses, * n* (%)	78 (36.6%)	40 (40%)
*S. aureus* colonization test performed	100 (46.9%)	100 (100%)
*S. aureus* colonization, * n* (%)	44 (20.7%)	44 (44%)
Subglottic stenosis at diagnosis, * n* (%)	16 (7.5%)	6 (6%)
Subglottic stenosis at last visit, * n* (%)	17 (8.0%)	8 (8%)
Saddle nose at diagnosis, * n* (%)	11 (5.2%)	7 (7%)
Use of nasal steroids, * n* (%)^a^	80 (37.6%)	51 (51%)
Antibiotic treatment with positive *S. aureus*, * n* (%)^c^		28/44 (63.6%)
Cotrimoxazole		22/44 (50%)
Azithromycin		2/44 (4.5%)
Mupirocin ointment		17/44 (38.6%)
Unknown		4/44 (9.1%)
Antibiotic treatment with negative *S. aureus*, * n* (%)^d^		12/56 (21.4%)
Cotrimoxazole		10/56 (17.9%)
Azithromycin		0/56 (0%)
Mupirocin ointment		11/56 (19.6%)

Values are median (interquartile range IQR) or * n* (%), P value ≤ 0.05

*BVAS3* Birmingham vasculitis activity score version 3, *ANCA* anti-neutrophilic cytoplasmic autoantibody, *AAV* ANCA-associated vasculitis, *ENT* ear nose and throat

^a^Defined as receiving at least one prescription of nasal steroids during follow-up

^b^Defined as receiving at least one prescription of antibiotic treatment during follow-up regardless of the outcome of a performed *S. aureus* colonization test

^c^Defined as receiving at least one prescription of antibiotic treatment during follow-up and the presence of at least one positive *S. aureus* colonization test during follow-up

^d^Defined as receiving at least one prescription of antibiotic treatment during follow-up and the absence of *S. aureus* colonization in a performed test during follow-up

**Fig. 1:**
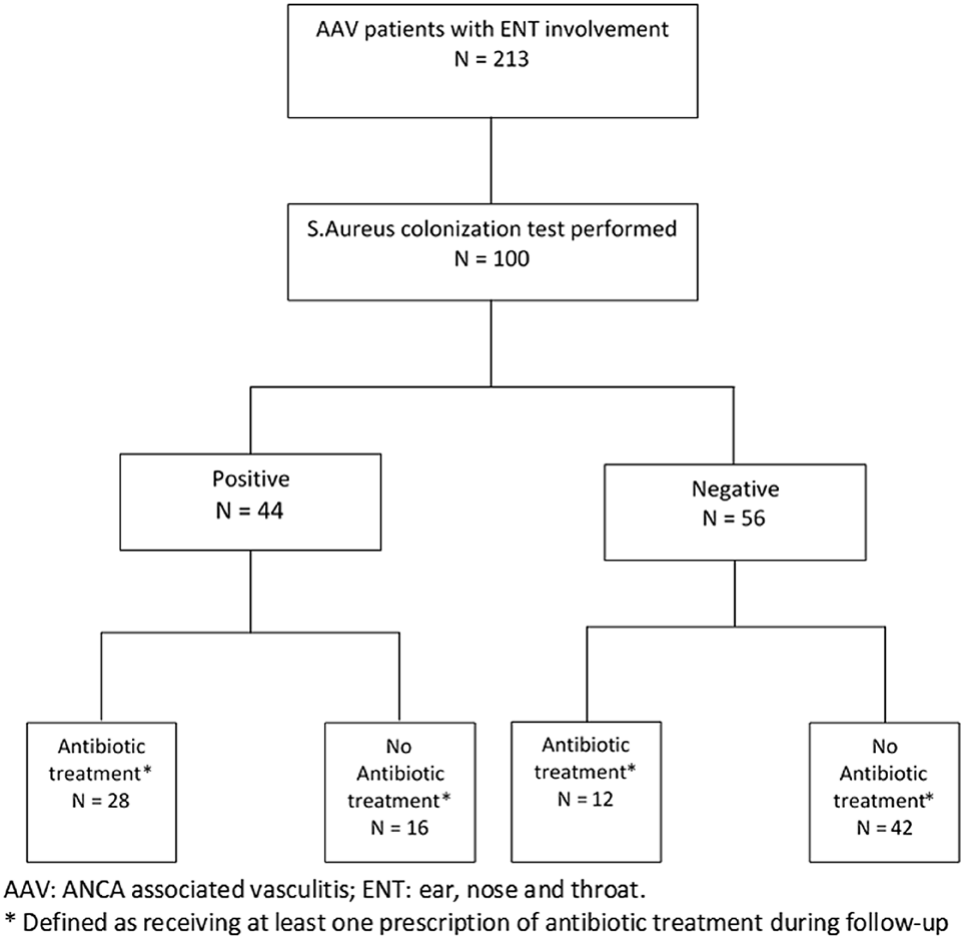
Flow chart of AAV patients with ENT involvement

*S. aureus* colonization was evaluated in 100 (46.9%) cases, of which 44 (44%) tested positive. Within the group of tested patients, antibiotics were prescribed to 40 (40%) patients regardless the presence of *S. aureus* colonization. A flow-chart is shown in Fig. 1. Cotrimoxazole was administered in 32 patients, azithromycin in two patients and 28 patients received nasal mupirocin ointment. In the patients who tested positive, 28 (28%) patients received antibiotic treatment. Dosages and duration of antibiotics varied amongst patients.

Relapse number per patient year was 0.1 (IQR 0–0.2) and the median BVAS3 score at last visit was 0 (IQR 0–4). Only one patient developed subglottic stenosis and 13 patients developed a saddle nose deformity during follow-up.

### Nasal* S. aureus* colonization and disease activity

There was no significant difference in systemic disease activity in patients with and without *S. aureus* colonization. The risk of relapse, relapse rate and BVAS3 at last visit were similar in patients with and without *S. aureus* colonization as shown in Table 2. With regard to local disease activity, 15 (50.0%) of the *S. aureus* colonized patients had at least one ENT relapse during follow-up compared to 25 (59.5%) of the *S. aureus* negative patients (*P* = 0.42). Four (11.4%) *S. aureus* colonized patients compared to three (6.4%) patients with a negative *S. aureus* test developed a saddle nose deformity (*P* = 0.42). Due to missing data not all 100 patients were included in each univariate analysis. For an overview of the number of included patients per each analysis, see supplementary table A.

**Table 2:** Disease activity of AAV patients with ENT involvement with or without *S. aureus* colonization

Disease activity	*S. aureus* colonization		*P* value
	Yes *n* = 44	No *n* = 56	
Systemic symptoms			
History of one or more relapses, * n* (%)	23 (54.8%)	34 (64.2%)	0.354
Relapse number per patient years, median (IQR)	0.09 (0–0.18)	0.12 (0–0.29)	0.191
BVAS3 score at last visit, median (IQR)	1 (0–4)	0 (0–4)	0.876
Local symptoms			
History of one or more ENT relapses, * n* (%)^a^	15 (50.0%)	25 (59.5%)	0.423
Development of saddle nose deformity during follow-up, * n* (%)^a^	4 (11.4%)	3 (6.4%)	0.419

Values are median (interquartile range IQR) or * n* (%)

*AAV* ANCA-associated vasculitis, *ANCA* anti-neutrophilic cytoplasmic autoantibody, *BVAS3* Birmingham vasculitis activity score version 3, *ENT* ear nose and throat

^a^More than 10% missing in analysis. For an overview of the number of included patients per analysis, see supplementary table A

Regression analysis showed that when corrected for gender, age at onset, AAV type, follow-up time and use of nasal steroids, there was no difference in relapse number per patient year between AAV patients colonized with *S. aureus* versus non-colonized patients (RR 2.03; *P* = 0.06) as shown in Table 3. Also, no difference was found in ENT relapses (OR 0.13; *P* = 0.14) and saddle nose deformity (OR 0.61; *P* = 0.74) between patients with and without *S. aureus* colonization.

**Table 3:** Risk of active disease in *S. aureus* colonized patients

Disease activity	OR/RR	95% CI	*P* value
Systemic symptoms (risk ratio)			
Relapse number per patient years	2.03	0.97–4.26	0.06
Local symptoms (odds ratio)			
History of one or more ENT relapses	0.13	0.06–1.47	0.14
Development of saddle nose deformity during follow-up	0.61	0.04–10.68	0.74

Values are presented as odds ratio’s or risk ratio’s with a 95% confidence interval

*ENT* ear nose and throat

### Antibiotic treatment and disease activity

Of the 44 patients with *S. aureus* colonization, 28 (63.6%) patients received antibiotics aimed to eradicate *S. aureus*. In the 56 patients without *S. aureus* colonization, 12 (21%) received antibiotics. No data was available concerning antibiotic use from four of 44 *S. aureus* colonized patients.

No statistically significant difference in systemic and local disease activity was found between AAV patients colonized with *S. aureus* that received antibiotics for *S. aureus* eradication compared with patients colonized with *S. aureus* not receiving these antibiotics, as shown in Table 4. The number of patients was too small for regression analysis. Due to missing data not all 40 patients were included in each univariate analysis. For an overview of the number of included patients per each analysis, see Supplementary table B.

**Table 4:** Effect of antibiotics on disease activity in patients with ENT involvement and *S. aureus* colonization

Disease activity	Antibiotic treatment		*P* value
	Yes (*n* = 28)	No (*n* = 12)	
Systemic symptoms			
History of one or more relapses, * n* (%)	18 (47.4%)	4 (10.5%)	0.635
Relapse number per patient years, median (IQR)^a^	0.11 (0–0.18)	0.17 (0.02–0.26)	0.346
BVAS3 last visit, median (IQR)	1 (0–4)	1 (0–4)	0.932
Local symptoms			
History of one or more ENT relapses, * n* (%)^a^	9 (33.3%)	3 (11.1%)	0.438
Development of saddle nose deformity during follow-up, * n* (%)^a^	4 (12.1%)	0 (0%)	0.367

Values are median (interquartile range IQR) or * n* (%)

*AAV* ANCA-associated vasculitis, *ANCA* anti-neutrophilic cytoplasmic autoantibody, *BVAS3* Birmingham vasculitis activity score version 3, *ENT* ear nose and throat. Antibiotic treatment is defined as at least one prescription of cotrimoxazole, azithromycin and/or mupirocin aimed at *S. aureus* eradiation

^a^More than 10% missing in analysis. For an overview of the number of included patients per analysis, see supplementary table B

## Discussion

Our study shows no difference in systemic and local disease activity between the patients colonized with *S. aureus* and *S. aureus* negative patients. Neither did we observe an effect of antibiotics on local and systemic disease activity.

These outcomes are in line with results from a prospective observational cohort study by Tan et al. [13]. In this study, adult AAV patients were observed for 4 years. No association was found between nasal *S. aureus* colonization and the extent of symptoms. Low-dose cotrimoxazole (dosage 400–80 mg/day), achieved less nasal *S. aureus* colonization over time. However, no effect on AAV evolution was observed.

In contrast to our findings, Salmela et al. did find an association between v and disease activity suggesting that *S. aureus* could play a role in triggering more active GPA [10]. In a prospective multicentre survey study (SAVAS), based on two randomized controlled trials, a significant association between chronic *S. aureus* nasal colonization and relapse rate in generalized AAV and in early systemic AAV was observed. Chronic nasal *S. aureus* colonization was almost exclusively seen in GPA patients and therefore only GPA patients were included. No data from MPA or EGPA patients were included in the analyses.

It is possible that *S. aureus* is found more often in patients with chronically active ENT disease as a result of local damage, creating an opportunity for *S. aureus* to colonize patients. In this scenario chronic *S. aureus* colonization is a result of active disease rather than a causative factor. This would also explain why a single positive swab does not necessarily has to relate with disease activity, as was found in our and other studies [13, 24].

Also, the absence of difference between the *S. aureus* positive and negative group in disease activity, could indicate that *S. aureus* may play only a minor pathogenic role. Research from Rhee et al. examined nasal microbiota (bacteria and fungi) in GPA patients and compared this to healthy controls [25]. They found that GPA patients compared to healthy individuals, had a significantly different microbial composition and had dysbiosis in the nose resulting in a lower prevalence of Propionibacterium acnes and Staphylococcus epidermidis which both compete with *S. aureus* [26, 27]. However they found no difference in the abundance of *S. aureus* between GPA and controls in contrast to previous studies [28, 29]. Rhee et al. suggested that manipulation of the nasal microbiome could be a novel therapeutic target [25]. This could mean that the role of solely *S. aureus* colonization in AAV pathophysiology is smaller than assumed.

With regard to antibiotic use, we found no beneficial effect on disease activity. According to Salmela et al. the use of cotrimoxazole treatment in low doses did not influence the relapse risk even though the rate of chronic nasal *S. aureus* colonization was almost completely prevented [10]. The absence of effect of antibiotics on disease activity is in line with our findings as well as the findings of Tan et al. but in strong contrast with Stegeman et al. who performed a prospective, randomized, placebo-controlled study to evaluate the efficacy of high dose cotrimoxazole in preventing relapses in GPA patients [9]. This study consisted of a group of 41 patients receiving cotrimoxazole and 40 patients received placebo. GPA patients with and without ENT involvement were enrolled. They found that cotrimoxazole in a dosage of 960 mg twice daily for a period of 24 months, reduced the incidence of ENT relapses in GPA patients and the use of cotrimoxazole was identified as a factor related to disease free interval. A potential explanation for the lack of effect of antibiotics on disease activity in our study and others could be the lower dosages used [10, 13, 21]. In our study different dosages and duration of antibiotics were used, and a small group of patients used the higher dosage of 960 mg of cotrimoxazole twice daily. Unfortunately, subgroup analysis was not possible due to small group size. Furthermore, patients included in the randomized, placebo-controlled trial from Stegeman et al. were patients with GPA in remission in contrast to our study were no difference was made in remission or active disease.

Limitations of our study include the small number of patients tested for *S. aureus* colonization and the small group of patients receiving antibiotic treatment. Due to the retrospective design of our study, there were missing data on duration of antibiotic treatment and confounding by indication could have occurred. Also, some patients did not receive antibiotic treatment aimed at *S. aureus* colonization but did receive cotrimoxazole as prophylaxis for Pneumocystis jiroveci-pneumonia. This could have been a confounder not being corrected for. Lastly, we defined *S. aureus* colonization as at least one positive test during follow-up time, therefore no differentiation between intermittent and chronic carriers could be made.

In conclusion, in this retrospective cohort study in AAV patients with ENT involvement, no difference was found in local and systemic disease activity between patients with and without nasal *S. aureus* colonization. In case of nasal *S. aureus* colonization, antibiotic treatment did not influence local or systemic disease activity. It is therefore possible that *S. aureus* plays a smaller role in AAV than previously thought. The role of antibiotic treatment in AAV patients colonized with *S. aureus* on AAV disease activity needs to be prospectively evaluated in a larger cohort.

## Supplementary Information

Below is the link to the electronic supplementary material.

The effect of nasal Staphylococcus aureus colonization and antibiotic treatment on disease activity in ANCA-associated vasculitis1 (DOCX 19 KB)
