# Supplementary material for: The effect of nasal Staphylococcus aureus colonization and antibiotic treatment on disease activity in ANCA-associated vasculitis: a retrospective cohort study in the Netherlands
Source: Rheumatol Int. 2022 Oct 26;43(3):467–75. doi: 10.1007/s00296-022-05228-8 (PMC9968256; doi:10.1007/s00296-022-05228-8)
Supplement: Supplementary file 1 — The effect of nasal Staphylococcus aureus colonization and antibiotic treatment on disease activity in ANCA-associated vasculitis1 (DOCX 19 KB) [file 296_2022_5228_MOESM1_ESM.docx]

Supplementary tables

| **Supplementary table A: Overview of included AAV patients with ENT involvement with or without S. aureus colonization in each analysis per disease activity** | | | |
| --- | --- | --- | --- |
| **Disease activity** | **Data (n=100)** | | |
|  | **S. aureus colonization** | | **Missing** |
|  | **Yes (n=44)** | **No (n=56)** |  |
| Systemic symptoms | | | |
| History of one or more relapses, n(%) | 42 (42%) | 53 (53%) | 5 (5%) |
| Relapse number per patient years, n(%) | 40 (40%) | 52 (52%) | 8 (8%) |
| BVAS3 score at last visit, n(%) | 42 (42%) | 56 (56%) | 2 (2%) |
| Local symptoms | | | |
| History of one or more ENT relapses, n(%) | 30 (30%) | 42 (42%) | 28 (28%) |
| Development of saddle nose deformity during follow-up, n(%) | 35 (35%) | 47 (47%) | 18 (18%) |
| Development of subglottic during follow-up, n(%) | 41 (41%) | 51 (51%) | 8 (8%) |
| *Values are as n (%). AAV: ANCA associated vasculitis; ANCA:* *Anti-Neutrophilic Cytoplasmic Autoantibody; BVAS3: Birmingham Vasculitis Activity Score version 3; ENT: ear, nose and throat.* | | | |

| **Supplementary table B: Overview of included AAV patients with ENT involvement and positive S. aureus colonization with or without a history of antibiotic treatment in each analysis per disease activity** | | | |
| --- | --- | --- | --- |
| **Disease activity** | **Data (n=40)** | | |
|  | **Antibiotic treatment** | | **Missing** |
|  | **Yes** | **No** |  |
| Systemic symptoms | | | |
| History of one or more relapses, n(%) | 32 | 6 | 2 |
| Relapse number per patient years, n(%) | 31 | 5 | 4 |
| BVAS3 last visit, n(%) | 32 | 6 | 2 |
| Local symptoms | | | |
| History of one or more ENT relapses, n(%) | 22 | 5 | 13 |
| Development of saddle nose deformity during follow-up, n (%) | 28 | 5 | 7 |
| Development of subglottic during follow-up, n(%) | 31 | 6 | 3 |
| *Values are as n (%). AAV: ANCA associated vasculitis; ANCA:* *Anti-Neutrophilic Cytoplasmic Autoantibody; BVAS3: Birmingham Vasculitis Activity Score version 3; ENT: ear, nose and throat. Antibiotic treatment is defined as at least one prescription of cotrimoxazole, azithromycin and/or mupirocin aimed at S. aureus eradiation.* | | | |
